# The potential of mHealth for older adults on dialysis and their care partners: What’s been done and where do we go from here?

**DOI:** 10.3389/fneph.2022.1068395

**Published:** 2023-01-06

**Authors:** Brett Burrows, Nicole DePasquale, Jessica Ma, C. Barrett Bowling

**Affiliations:** ^1^ Center for the Study of Aging and Human Development, Department of Medicine, Duke University School of Medicine, Durham, NC, United States; ^2^ Division of General Internal Medicine, Department of Medicine, Duke University School of Medicine, Durham, NC, United States; ^3^ Geriatric Research, Education, and Clinical Center, Durham Veteran Affairs Health Care System (VAHCS), Durham, NC, United States; ^4^ Center of Innovation to Accelerate Discovery and Practice Transformation, Durham VACHS, Durham, NC, United States; ^5^ Department of Biostatistics and Bioinformatics, Duke University School of Medicine, Durham, NC, United States

**Keywords:** end-stage kidney disease (ESKD), dialysis, mobile health (mHealth), care partner, older adults, self-care, patient engagement, stakeholder involvement

## Abstract

Self-care, or the dynamic, daily process of becoming actively involved in one’s own care, is paramount to prevent and manage complications of end-stage kidney disease. However, many older dialysis patients face distinctive challenges to adequate engagement in self-care. One promising strategy for facilitating self-care among older dialysis patients and their care partners is the utilization of mobile health (mhealth). mHealth encompasses mobile and wireless communication devices used to improve healthcare delivery, patient and care partner outcomes, and patient care. In other disease populations, mHealth has been linked to maintenance of or improvements in self-management, medication compliance, patient education, and patient-provider communication, all of which can slow disease progression. Although mHealth is considered feasible, acceptable, and clinically useful, this technology has predominately targeted younger patients. Thus, there is a need to develop mHealth for older dialysis patients and their care partners. In this article, we describe current mHealth usage in older dialysis patients, including promising findings, challenges, and research gaps. Given the lack of research on mHealth among care partners of older dialysis patients, we highlight lessons learned from other disease populations to inform the future design and implementation of mHealth for these key stakeholders. We also propose that leveraging care partners represents an opportunity to meaningfully tailor mHealth applications and, by extension, improve care partner physical and mental health and decrease caregiver burden. We conclude with a summary of future directions to help older dialysis patients and their care partners receive recognition as target end-users amid the constant evolution of mHealth.

## Introduction

Chronic kidney disease (CKD) is a leading cause of morbidity and mortality worldwide (1). This progressive condition is characterized by substantial physical, psychological, and economic burdens; reductions in quality of life; and continuous management to delay the onset of end-stage kidney disease (ESKD) (2). In an effort to slow disease progression and prevent its complications, current CKD management guidelines consider self-care as a standard of care (3). Self-care is ideally defined as a dynamic, daily process in which the individual with CKD becomes actively involved in their own care through health-promoting and treatment adherence behaviors, monitoring signs and symptoms of the disease, and managing symptoms (4). The prospective benefits of self-care are numerous, including reducing patients’ disease burden and the development of morbidities, improving their quality of life and health outcomes, and decreasing the social, economic, and health care burden of chronic diseases (2). Nonetheless, self-care is multidimensional and complicated, and poor engagement has been well-documented among many individuals with CKD (3).

One predictor of inadequate engagement in CKD self-care behavior is older age, a trend attributed to older adults’ lower capacity for engagement relative to younger adults (5). Compared to younger counterparts, older adults face unique challenges in meeting personal care and support needs, such as multi-morbidity, functional limitations in activities of daily living, cognitive decline, frailty, socioeconomic disadvantage, social isolation, and geriatric conditions (6). For older adults with CKD who progress to ESKD, self-care becomes more complicated, as prescription medications, diet and fluid restrictions, and a healthy lifestyle are balanced with a more elaborate treatment plan involving dialysis treatment itself. The self-care difficulties of older adults is particularly concerning given that they demonstrate a striking decline in physical and mental aspects of quality of life in the year preceding dialysis initiation (7). These aforementioned difficulties, coupled with disease progression and the aging process, hinder the abilities of older adults with CKD and ESKD to adequately perform necessary self-care, creating an increasing dependence on the contributions of care partners to assist with self-care needs.

Care partners, or close family members and friends informally involved in ESKD management, have long been considered health care providers’ greatest allies in treating older adults on dialysis. Care partners are tasked with navigating a complex role that usually entails providing myriad forms of self-care support, including transportation to and from dialysis and medical appointments; assistance with dietary prescription and meal preparation; administration of medication; and observation of changes in health and functional status (8). Their support is associated with several beneficial patient outcomes, such as improved self-management behaviors, compliance and psychosocial adjustment to treatments, and quality of life, as well as decreased mortality risk, treatment complications, anxiety and depressive symptoms, and odds of 30-day hospital readmission (9–13). Although their active participation in treatment can be rewarding, care partners report related burdens, adverse effects on health and well-being, and conflicts with other roles and responsibilities (14). Further exacerbating matters is care partners’ persistent oversight in research and in practice. Predictably, care partners convey uncertainty about their role in self-care, describe feeling overlooked by health care providers, encounter difficulties accessing the health care system, lack disease- and treatment-related knowledge, and report unmet support needs (15). Given that care partners affect and are affected by dialysis treatment, it is imperative to identify opportunities to acknowledge, meaningfully support, and better integrate their influential participation.

One potentially promising strategy for simplifying the complexity of and facilitating self-care among older dialysis patients and care partners alike is the incorporation of mobile health (mHealth). There is no single, universally agreed upon definition of mHealth at this time. For our purposes, we use the term mHealth to encompass mobile and wireless communication devices (e.g., text messaging, mobile apps, and/or wearable devices) intended to be user-friendly tools to improve healthcare delivery, patient and care partner outcomes, and impact patient care beyond conventional clinical care; we consider mHealth to be a subset of telehealth, as telehealth is a much broader term reflecting the use of digital information (e.g., electronic health records) and communication technologies for healthcare. Despite a lack of consensus on its meaning, mHealth has emerged as an essential model of care due, in part, to the expedited and large-scale uptake of virtual or remote health care delivery necessitated by the varied impacts of the COVID-19 pandemic (16, 17). The increasing popularity and usage of mHealth has become evident in recent disease management programs for chronic conditions such as diabetes, hypertension, and cardiovascular disease (18–20). These programs have highlighted several benefits associated with the incorporation of mHealth, including the promotion of key self-management principles; improvements in patient-provider communication and clinical outcomes such as blood pressure, blood sugar, and pain management; enhanced patient autonomy, activation, health status, knowledge, and self-efficacy; and reduced health care utilization (21). Yet, few mHealth applications are tailored to the ESKD population, particularly among older adults and their care partners. This paper describes the fledgling use of mHealth strategies to support self-care in older dialysis patients and their care partners. Finally, we present future directions for supporting stakeholder involvement and tailoring devices to the specific needs of older dialysis patients and their care partners to better leverage mHealth’s capabilities.

## mHealth and Dialysis

To date, few clinical trials have investigated the potential impact of mHealth on patients receiving dialysis. These trials have mainly focused on improving lifestyle behavior changes through remote or self-monitoring interventions that primarily entailed the use of smartphone apps, tablets, or personal digital assistants to deliver content (22, 23). Though limited mHealth-related trials exist, patients with ESKD overwhelmingly report high levels of satisfaction and willingness to continue using mHealth devices, indicating high acceptability and feasibility for self-care management and clinical use (22, 23). Importantly, mHealth has also impacted the economic burden on patients receiving dialysis, healthcare providers, and the healthcare system. Trials assessing economic impact have consistently concluded that remote monitoring led to significant healthcare cost savings given decreased healthcare resource utilization and reduced medication and hospitalization costs (22). Despite mHealth showing benefits in patient-reported outcomes and economic impact, many trials have resulted in non-significant improvements for health outcomes (e.g., blood pressure, ultrafiltration rate, blood profile, and interdialytic weight gain). Few trials, combined with small sample sizes, short duration, and non-significant improvements in health outcomes, begs the question of whether mHealth is clinically effective. Nonetheless, mHealth has shown great potential for improving patient reported outcome measures (PROMs) and conventional clinical care through greater patient self-care engagement.

In **Table 1**, we categorized the potential impacts of mHealth and conventional clinical care into four major themes (economic, clinical, person-centered, and accessibility) and highlighted significant potential advantages and disadvantages of each. Emphasized is mHealth’s overarching advantage to impact clinical and person-centered outcomes by engaging patients in their own care through self-monitoring, disease education, personalization/cultural tailoring, and real-time updates (22). Yet, under current conventional clinical care, low patient engagement is far too common in the dialysis setting. Critical to these low levels of patient engagement is the lack of personalized care and personalized/culturally tailored resources (self-care and educational). Many patients feel they are not knowledgeable enough to make informed decisions and they lack the resources to better care for themselves (24). mHealth can empower patients by providing resources and unlimited personalization and cultural tailoring, as well as disease-related education to enhance self-efficacy for greater self-care management. mHealth can also monitor changes in PROMs and give real-time updates to healthcare providers for more timely care. In a recent study, Viecelli et al. (25) highlighted the effective symptom monitoring ability of mHealth by demonstrating that real-time updates on PROMs resulted in prompt and efficient discussion, resulting in active engagement of patients and healthcare provider(s). mHealth’s capability to provide immediate evaluation of PROMs is in stark contrast to the delayed feedback of current clinical practice. Presently, paper surveys are the primary method of measuring and assessing PROMs. This method is resource intensive, generates survey fatigue, and limits optimal care because feedback can be delayed for months.

**Table 1:** Potential advantages and disadvantages with conventional clinical care vs mobile health (mHealth) technology.

Impact	Conventional Clinical Care		mHealth Technology	
	Proposed advantages	Proposed disadvantages	Proposed advantages	Proposed disadvantages
**Economic**	Insurance coverage for in-person visits	Time, travel/transportation, and cost to obtain in person care	Time and cost efficient (e.g., transportation costs, reduced hospitalizations)	Out-of-pocket costs of mHealth equipment or technology (e.g., mobile applications)
**Clinical**	Face-to-face care	Limited focus on patient reported outcomes measures (PROMs)	Social distancing during the COVID-19 pandemic	Limited physical examination
	Supervised care*	Resource intensive (potential for delayed feedback and care)	Potential for improved clinical outcomes (e.g., real-time updates)	Unsupervised care* (safety concerns)
			Impact to clinical workflow (e.g., change in workflow patterns, time management, and unreliable internet services)	
**Person-Centered**	In-person interaction with clinical team	High patient and care partner burden^	Potential to alleviate patient and care partner burden^ (e.g., set medication reminders)	Loss of more personal interaction with clinical team
		Variable patient and care partner engagement (e.g., participation in care)	Facilitate patient and care partner engagement	
		Lack cultural tailoring	Cultural tailoring (e.g., specific language settings)	
		Interpreter for non-English speakers		
		Resources designed for the general dialysis patient	Personalized resources for improved self-care/decision making	
**Accessibility**	Access does not depend on technology availability and/or literacy	Patient data limited by intermittent patient visits	Real-time remote access to patient data	Technology literacy affects use
			Potential large-scale dissemination	Privacy or security concerns (e.g., data breach)
				Digital divide (wireless connectivity, advanced technology)

*Supervised care is care delivered by any health care provider (e.g., nurses, therapists, social workers, advance practice practitioners, and/or physicians).

^Patient/care partner burden relates to reliance on the patient/care partner to perform necessary tasks as part of their self-care.

While timely care can be facilitated by mHealth, remote care practices pose disadvantages to clinical practice and person-centered outcomes. For many patients with ESKD, these disadvantages are largely due to limited regular physical examinations, face-to-face interaction, and accessibility of advanced technology. mHealth characteristics restrict personal connection with healthcare provider(s), which is imperative for dialysis care. There are also major concerns over privacy and security. Limited regulations currently constrain developers of mHealth apps to prevent lost or stolen data, thereby increasing patients’ reluctance to use and adopt mHealth for self-care support. Moreover, major issues revolve around accessibility. While the economic advantages of mHealth are potentially widespread (e.g., reduced healthcare costs and/or patient travel expenses), advanced technology is costly and not universally accessible. The high cost of advanced technology prevents adoption, ultimately resulting in low technology literacy. Collectively, high cost and low technology literacy contribute to the digital divide.

The digital divide may, in part, limit dissemination of mHealth within dialysis centers. Many patients, especially older adults on dialysis, are hesitant to use and participate in mHealth trials because of perceived low technology literacy fueled by accessibility issues, inadequate health literacy, and complications with disease-related symptoms. For example, older adults on dialysis are at greater risk of co-morbidity, frailty, and cognitive impairment; all of which are independently associated with lower adoption of technology and make navigating technological devices (e.g., smartphones) more difficult. In addition, lower educational attainment and health and technology literacy limit their ability to fully benefit from mHealth. Despite these barriers, positive interest in using mHealth devices for self-care expressed by patients receiving dialysis, including older adults, suggests they are willing and able to participate in mHealth-related research (26). However, such research has largely neglected to address barriers encountered by older adults on dialysis, resulting in ongoing suboptimal participation in mHealth trials (26, 27). Thus, there remains a major knowledge gap in how mHealth affects self-care behaviors and outcomes. It is imperative for stakeholders to generate ways to leverage the interest of and mitigate barriers for older adults on dialysis when developing and testing mHealth technologies.

## mHealth and Care partners

Consistent with care partners’ relative invisibility in dialysis-related research and care, their participation is seldom accounted for in the technology-aided delivery of self-care support to chronic disease patients. Likewise, mHealth available in ESKD has rarely focused on care partners as the primary users or target audience. To address these knowledge gaps, lessons learned from mHealth for care partners of older adults and patients with other diseases can be applied to the design and implementation of mHealth for care partners of patients with ESKD. Two disease populations that have paved the way for mHealth research with care partners include dementia and stroke. Because self-care for patients with these conditions have a similar reliance on care partners and may contribute to care partner burden, interventions from these populations are likely suitable for adaptation to care partners of patients with ESKD. These studies have shown that mHealth interventions can improve care partner physical and mental health, increase care partner self-efficacy, and decrease caregiver burden. For example, mHealth can decrease out-of-pocket healthcare costs by allowing care partners to remotely access resources otherwise limited by travel time, distance, or mobility (28). Generally, care partner-oriented mHealth programs have fallen into three domains: 1) Building relationships (social support, services, resources), 2) care support for the patient (education, remote monitoring, and patient care), and 3) support for the care partner (therapy, self-care) (28, 29). Technologies from these populations potentially applicable for care partners of patients with ESKD include communication with the healthcare team and family, reminder systems (e.g., appointments and medications) and symptom monitoring, and care partner education and support (30, 31). These promising, existing interventions from other disease populations present an opportunity for mHealth developers to build upon this work and tailor to the specific needs of ESKD care partners.

Additionally, key stakeholders and developers of mHealth for care partners need to be aware of known barriers that limit adoption of mHealth. Adoption of mHealth is affected by accessibility, small print size, lack of health and technology literacy, lack of subtitles for the hearing impaired, and high cost, all of which impede care partner engagement (28). Furthermore, care partners may need to install and juggle multiple applications to meet the needs of the patient and themselves because most mobile applications for care partners only address one function (31). Limited accessibility and functionality impact overall utilization of mHealth in care partners and may be due to the lack of user input from care partner stakeholders. Thus, we suggest care partners be involved as both key stakeholders as well as end-users in mHealth-related research.

## Future implications

**Figure 1:**
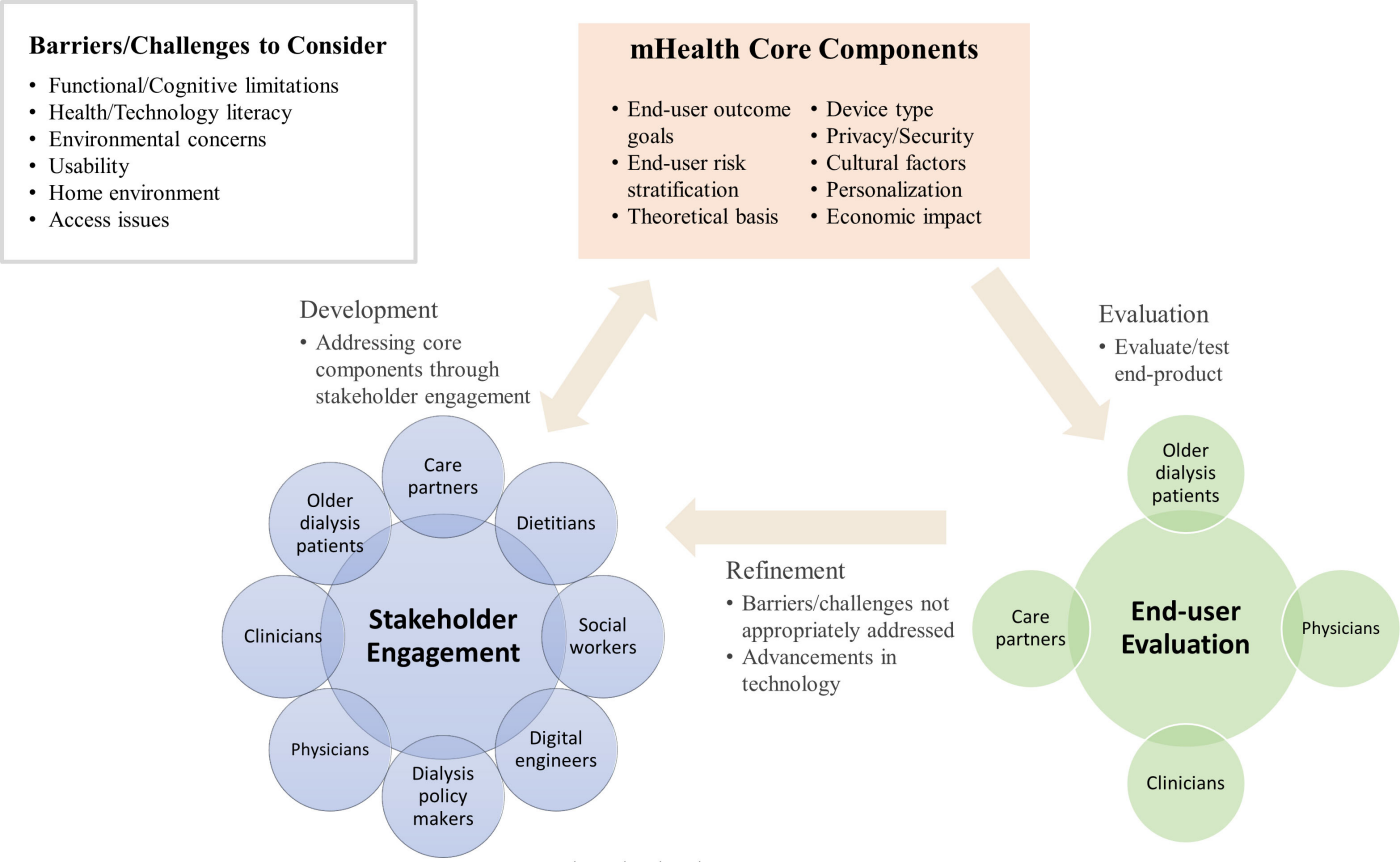
Conceptual model for a person-centered approach to the development of mHealth for older adults on dialysis and their care partners.

Although much work has been done in recent years to keep pace with the unprecedented growth and subsequent use of mHealth, much work remains to be done. To date, there is no one definition of mHealth and confusion centers around associated terms and various technology platforms. Terms like telehealth, telemedicine, electronic health, wearable technology, and digital health are commonly used interchangeably when in fact there are slight nuances to each. Establishing distinct definitions may help alleviate barriers and expand the integration of these devices into healthcare and the older dialysis population. We therefore recommend the use of a multidisciplinary team of key stakeholders (e.g., physicians, clinicians, patients, care partners, social workers, digital engineers, dialysis organization representatives, etc.) to collectively work together to successfully address the barriers and challenges (i.e., safety, utility, accessibility, and literacy) of mHealth for older dialysis patients. Furthermore, we call for a common framework criterion (**Figure 1**) for the development of mHealth-related research. Identifying a common framework can propel the developmental stages to keep pace with technology advancements and reduce improper use of mHealth devices by end-users. Integrating these efforts into the development of mHealth may help mitigate symptom burden and for some, delay the progression from CKD to dialysis initiation. Supporting these proposed future directions, the Patient-Centered Outcomes Research Institute and Kidney Health Initiative have set forth recent initiatives aimed at improving patient-centered care and outcomes through engagement of key stakeholders, including patients, care partners, and other patient advocates, in the development and dissemination of patient-centered research. These initiatives offer encouragement for the future of mHealth in CKD as more trials and larger sample sizes are needed to better determine mHealth’s clinical effectiveness and safety among older adults receiving dialysis.

## Patients

Not only are older dialysis patients infrequently included in mHealth trials, but they are also largely excluded as key stakeholders in the development of mHealth supposedly intended for their own use. The majority of mHealth research trials (and especially commercially available apps) have been created without input from patients receiving dialysis. Lack of patient stakeholder involvement fails to address the specific needs of patients on dialysis and may contribute to mixed findings regarding clinical outcomes. Inclusion of older adults as key stakeholders in the development of mHealth is a crucial element missing in dialysis-related research and may serve as an opportunity to educate patients with ESKD on the potential advantages of mHealth devices, empowering them to improve self-care and/or to make more informed decisions about best care options. If older dialysis patients feel more knowledgeable using these devices, they will exhibit greater self-efficacy and be more likely to adopt them as part of their own self-care routine. Likewise, inclusion of older dialysis patients as key stakeholders can also make them feel more valued, which can be empowering (32) and subsequently improve psychological well-being. However, adopting mHealth may be impossible for many older adults on dialysis because of physical and/or cognitive impairments. For these patients, inclusion of care partners is essential. Incorporating care partners as key stakeholders may serve to improve the utility and effectiveness of mHealth for patients receiving dialysis given that they are uniquely positioned to provide invaluable insight into the real-world context in which ESKD self-care practices occur.

## Care partners

We propose that mHealth offers at least two promising opportunities to acknowledge, meaningfully support, and better integrate the influential participation of care partners in ESKD self-care. One opportunity involves the inclusion of care partner perspectives in the development of self-care technology designed for older adults on dialysis. Understanding these important stakeholder perspectives may facilitate care partner engagement, increase the relevance of self-care content, help address barriers to older adults’ mHealth use, improve recognition of older adults’ home or social contexts, and enable care partners to feel that their insight, experiences, and opinions are valued.

An additional opportunity entails targeting care partners as users of mHealth. The care partner role requires ongoing and adaptive support in light of changing care recipient needs and varied impacts on daily life over time. Tailoring mHealth specifically for care partners may alleviate uncertainty about their role in dialysis care, foster empowerment, improve disease- and treatment-related knowledge, mitigate related burdens, and address unmet support needs. mHealth strategies that directly appeal to care partners’ individual needs are also critical because of dyadic interdependences in the relationship between older adults and care partners. That is, care partner health and well-being can affect patient health and well-being (and vice versa). Thus, mHealth capable of positively impacting the care partner can, in turn, positively impact the patient. Accordingly, we recommend that the design and implementation of mHealth technologies focus on the care partner as an end-user and incorporate usability, feasibility, and acceptability testing (28, 31, 33). However, these promising opportunities will continue to reflect missed opportunities until care partner participation is fully realized as a resource that can be leveraged to promote mHealth adoption and, in turn, support care partner participation and patient self-care.

## Conclusion

mHealth has demonstrated great promise in improving self-care practices of several chronic conditions and offers the potential to support self-care, improve patient activation, and promote health and well-being among older patients with ESKD and their care partners. In order for older dialysis patients and their care partners to likewise participate and benefit from mHealth interventions, these key stakeholders must be incorporated into relevant clinical trials, their experiences and perspectives must be accounted for in the creation and design of self-care support, and their individual person-centered needs, as well as those of the patient-care partner dyad as a whole, should be prioritized. Additionally, a greater emphasis should be placed on collaboration between physicians, clinicians, patients, care partners, and anyone else involved in the care of the dialysis patient. Collaborations need to be continual and consistent to reassess strategies to offset the unpredictable health trajectory patients and care partners navigate, as well as the constant evolution of mHealth. Until these collaborations are built and patients’ and care partners’ voices are heard, mHealth’s potential to improve patient outcomes will be limited.

## Data availability statement

The original contributions presented in the study are included in the article/**Supplementary Material**. Further inquiries can be directed to the corresponding author.

## Author contributions

All authors listed have made a substantial, direct, and intellectual contribution to the work and approved it for publication.

## References

[1] Carney EF . The impact of chronic kidney disease on global health. Nat Rev Nephrol (2020) 16(5):251. doi: 10.1038/s41581-020-0268-7 32144399

[2] Centers for Disease Control and Prevention (CDC) . Chronic kidney disease in the united states, 2021. Atlanta, GA: US Department of Health and Human Services, Centers for Disease Control and Prevention (2021).

[3] Schrauben SJ Rivera E Bocage C Eriksen W Amaral S Dember LM . A qualitative study of facilitators and barriers to self-management of CKD. Kidney Int Rep (2021) 7:46–55. doi: 10.1016/j.ekir.2021.10.021 35005313 PMC8720654

[4] Jaarsma T Strömberg A Dunbar SB Fitzsimons D Lee C Middleton S . Self-care research: How to grow the evidence base? Int J Nurs Stud (2020) 105:103555. doi: 10.1016/j.ijnurstu.2020.103555 32199150

[5] Almutary H Tayyib N . Factors influencing self-management among non-dialysis chronic kidney disease patients. Healthcare (Basel Switzerland) (2022) 10(3):436. doi: 10.3390/healthcare10030436 35326914 PMC8954207

[6] Schrauben SJ Hsu JY Wright Nunes J Fischer MJ Srivastava A Chen J . Health behaviors in younger and older adults with CKD: Results from the CRIC study. Kidney Int Rep (2019) 4(1):80–93. doi: 10.1016/j.ekir.2018.09.003 30596171 PMC6308910

[7] Kurella Tamura M Covinsky KE Chertow GM Yaffe K Landefeld CS McCulloch CE . Functional status of elderly adults before and after initiation of dialysis. New Engl J Med (2009) 361(16):1539–47. doi: 10.1056/NEJMoa0904655 PMC2789552 19828531

[8] Hoang VL Green T Bonner A . Informal caregivers' experiences of caring for people receiving dialysis: A mixed-methods systematic review. J Renal Care (2018) 44(2):82–95. doi: 10.1111/jorc.12235 29357407

[9] Chen YC Chang LC Liu CY Ho YF Weng SC Tsai TI . The roles of social support and health literacy in self-management among patients with chronic kidney disease. J Nurs Scholarship an Off Publ Sigma Theta Tau Int Honor Soc Nursing (2018) 50(3):265–75. doi: 10.1111/jnu.12377 29569423

[10] Flythe JE Hilbert J Kshirsagar AV Gilet CA . Psychosocial factors and 30-day hospital readmission among individuals receiving maintenance dialysis: A prospective study. Am J Nephrol (2017) 45(5):400–8. doi: 10.1159/000470917 PMC5483850 28407633

[11] Gerogianni G Babatsikou F Polikandrioti M Grapsa E . Management of anxiety and depression in haemodialysis patients: the role of non-pharmacological methods. Int Urol Nephrol (2019) 51(1):113–8. doi: 10.1007/s11255-018-2022-7 30456545

[12] Kim K Kang GW . The quality of life of hemodialysis patients is affected not only by medical but also psychosocial factors: a canonical correlation study. J Korean Med Sci (2018) 33(14):e111. doi: 10.3346/jkms.2018.33.e111 29607636 PMC5879041

[13] Untas A Thumma J Rascle N Rayner H Mapes D Lopes AA . The associations of social support and other psychosocial factors with mortality and quality of life in the dialysis outcomes and practice patterns study. Clin J Am Soc Nephrol CJASN (2011) 6(1):142–52. doi: 10.2215/CJN.02340310 PMC3022236 20966121

[14] DePasquale N Cabacungan A Ephraim PL Lewis-Boyér L Powe NR Boulware LE . Perspectives of African-American family members about kidney failure treatment. Nephrol Nurs J J Am Nephrol Nurses' Assoc (2020) 47(1):53–65. 32083437

[15] DePasquale N Cabacungan A Ephraim PL Lewis-Boyér L Powe NR Boulware LE . Family members' experiences with dialysis and kidney transplantation. Kidney Med (2019) 1(4):171–9. doi: 10.1016/j.xkme.2019.06.001 PMC7380377 32734197

[16] Schrauben SJ Appel L Rivera E Lora CM Lash JP Chen J . Mobile health (mHealth) technology: Assessment of availability, acceptability, and use in CKD. Am J Kidney Dis Off J Natl Kidney Foundation (2021) 77(6):941–50.e1. doi: 10.1053/j.ajkd.2020.10.013 PMC8154635 33309860

[17] Chan ASW Ho JMC Li JSF Tam HL Tang PMK . Impacts of COVID-19 pandemic on psychological well-being of older chronic kidney disease patients. Front Med (2021) 8:666973. doi: 10.3389/fmed.2021.666973 PMC8187602 34124096

[18] Li R Liang N Bu F Hesketh T . The effectiveness of self-management of hypertension in adults using mobile health: Systematic review and meta-analysis. JMIR mHealth uHealth (2020) 8(3):e17776. doi: 10.2196/17776 32217503 PMC7148553

[19] Gandhi S Chen S Hong L Sun K Gong E Li C . Effect of mobile health interventions on the secondary prevention of cardiovascular disease: Systematic review and meta-analysis. Can J Cardiol (2017) 33(2):219–31. doi: 10.1016/j.cjca.2016.08.017 27956043

[20] Wang Y Min J Khuri J Xue H Xie B A Kaminsky L . Effectiveness of mobile health interventions on diabetes and obesity treatment and management: Systematic review of systematic reviews. JMIR mHealth uHealth (2020) 8(4):e15400. doi: 10.2196/15400 32343253 PMC7218595

[21] Ong SW Jassal SV Porter E Logan AG Miller JA . Using an electronic self-management tool to support patients with chronic kidney disease (CKD): A CKD clinic self-care model. Semin Dialysis (2013) 26(2):195–202. doi: 10.1111/sdi.12054 23406283

[22] Yang Y Chen H . Intervention and evaluation of mobile health technologies in management of patients undergoing chronic dialysis: Scoping review. JMIR Mhealth Uhealth (2020) 8(4):e15549. doi: 10.2196/15549 32242823 PMC7165304

[23] Kosa SD Monize J D'Souza M Joshi A Philip K Reza S . Nutritional mobile applications for CKD patients: Systematic review. Kidney Int Rep (2019) 4(3):399–407. doi: 10.1016/j.ekir.2018.11.016 30899867 PMC6409338

[24] Woo K Pieters H . The patient experience of hemodialysis vascular access decision-making. J Vasc Access (2021) 22(6):911–9. doi: 10.1177/1129729820968400 PMC8216296 33118395

[25] Viecelli AK Duncanson E Bennett PN D'Antoine M Dansie K Handke W . Perspectives of patients, nurses, and nephrologists about electronic symptom monitoring with feedback in hemodialysis care. Am J Kidney Dis Off J Natl Kidney Foundation (2022) 80(2):215–26.e1. doi: 10.1053/j.ajkd.2021.12.007 35085687

[26] Hussein WF Bennett PN Pace S Chen S Legg V Atwal J . The mobile health readiness of people receiving in-center hemodialysis and home dialysis. Clin J Am Soc Nephrol (2021) 16(1):98–106. doi: 10.2215/CJN.11690720 PMC7792646 33355235

[27] Bowling CB Whitson HE Johnson TM 2nd . The 5Ts: Preliminary development of a framework to support inclusion of older adults in research. J Am Geriatrics Soc (2019) 67(2):342–6. doi: 10.1111/jgs.15785 PMC6532768 30693952

[28] Garnett A Northwood M . mHealth interventions to support caregivers of older adults: Equity-focused systematic review. JMIR Aging (2022) 5(3):e33085. doi: 10.2196/33085 35616514 PMC9308083

[29] Lobo EH Frølich A . mHealth applications to support caregiver needs and engagement during stroke recovery: A content review. Res Nurs Health (2021) 44(1):213–25. doi: 10.1002/nur.22096 33341958

[30] Kim E Baskys A Law AV Roosan MR Li Y Roosan D . Scoping review: the empowerment of alzheimer’s disease caregivers with mHealth applications. NPJ Digital Med (2021) 4(1):131. doi: 10.1038/s41746-021-00506-4 PMC8423781 34493819

[31] Grossman MR Zak DK . Mobile apps for caregivers of older adults: Quantitative content analysis. JMIR Mhealth Uhealth (2018) 6(7):e162. doi: 10.2196/mhealth.9345 30061093 PMC6090169

[32] Kalantar-Zadeh K Li PK-T Tantisattamo E Kumaraswami L Liakopoulos V Lui S-F . Living well with kidney disease by patient and care-partner empowerment: Kidney health for everyone everywhere. Am J Hypertension (2021) 34(2):220–5. doi: 10.1053/j.jrn.2021.01.024 33705539

[33] Matthew-Maich N Harris L . Designing, implementing, and evaluating mobile health technologies for managing chronic conditions in older adults: A scoping review. JMIR Mhealth Uhealth (2016) 4(2):e29. doi: 10.2196/mhealth.5127 27282195 PMC4919548

