# Supplementary material for: The potential of mHealth for older adults on dialysis and their care partners: What’s been done and where do we go from here?
Source: Front Nephrol. 2023 Jan 6;2:1068395. doi: 10.3389/fneph.2022.1068395 (PMC10479574; doi:10.3389/fneph.2022.1068395)
Supplement: Supplementary file 1 [file Table_1.docx]

**Supplementary Table 1.** Current state and proposed future directions of mHealth in end-stage kidney disease

|  | **Current State** | **Future Directions** |
| --- | --- | --- |
| **Defining mHealth** | - No universally agreed upon definition - Associated terminology used interchangeably despite nuance differences | - Use of multidisciplinary stakeholder team to establish distinct definitions for all terms - Development of a common framework criterion for mHealth-related research |
| **Stakeholder involvement in development of mHealth** | - Older adults on dialysis and their care partners are largely overlooked as key stakeholders - Digital divide remains a barrier to their involvement | - Leverage interest of and mitigate barriers to involvement of both parties - Capitalize on patient & care partner insight, especially for care recipients with physical or cognitive impairments - Take individual and dyadic approaches in the creation and design of mHealth |
| **Tailoring mHealth to care** | - Older adults on dialysis and their care partners are seldom targeted as users of mHealth | - Tailor mHealth specifically for older dialysis patients - Treat care partners as consumers/end-users of mHealth and tailor specifically to their needs |
| **Clinical trials** | - Few clinical trials have investigated the impact of mHealth on older adults on dialysis and their care partners - Clinical effectiveness remains in question - Small sample sizes - Short duration trials | - Conduct more clinical trials with older adults on dialysis and their care partners to better determine clinical effectiveness of mHealth and its varied impacts - Create such trials with input from key stakeholders - Larger sample sizes - Longer duration trials |
